# Approach to Liver Transplantation: Is There a Difference between East and West?

**DOI:** 10.3390/jcm13071890

**Published:** 2024-03-25

**Authors:** Nazli Begum Ozturk, Nathanial Bartosek, Merih Deniz Toruner, Aymen Mumtaz, Cem Simsek, Doan Dao, Behnam Saberi, Ahmet Gurakar

**Affiliations:** 1 Department of Internal Medicine, Corewell Health William Beaumont University Hospital, Royal Oak, MI 48073, USA; 2 Brown University Warren Alpert Medical School, Providence, RI 02903, USA; 3 Division of Gastroenterology and Hepatology, Johns Hopkins University, Baltimore, MD 21205, USA; 4 Division of Gastroenterology and Hepatology, Beth Israel Deaconess Medical Center, Harvard Medical School, Boston, MA 02215, USA

**Keywords:** cirrhosis, liver disease, liver failure

## Abstract

Liver transplantation (LT) remains the only curative treatment for end-stage liver disease as well as acute liver failure. With the exponential increase in organ demand due to the increasing incidence and prevalence of liver diseases, the need to overcome the supply and demand mismatch has arisen. In this review, we discuss the current universal status of LT, emphasizing various LT practices worldwide.

## 1. Introduction

The first successful liver transplantation (LT) was performed in 1967 by Dr. Thomas Starzl, with a congressional hearing approving LT for general use in 1983 [1]. LT is currently employed as a life-saving measure for multiple indications, including chronic liver disease, hepatocellular carcinoma (HCC), and acute liver failure [2,3]. In 2021, 34,694 LTs were performed globally, a 6.5% increase from 2020 and 20% increase from 2015 [2]. Two standard LT techniques applied by countries worldwide are living donor LT (LDLT) and deceased donor LT (DDLT). DDLT involves the transplantation of organs from legally deceased individuals and is the process employed for more than 90% of all LT activity in the Western world [2]. LDLT involves transplantation of a section of the liver, usually from family members, and constitutes most of the LT activity in many Asian countries, and it comprises 23% of all worldwide LTs [4,5]. Despite overall increasing numbers of DDLT and LLDT, the increasing burden of cirrhosis increases the need for LT globally, and efforts to expand the donor pool by using extended criteria donors, donations after circulatory death (DCD), ABO incompatible donors, machine perfusion, paired exchange or domino LT remain important [2].

## 2. Generalized Indications for Liver Transplantation

Etiologies for LT differ by region and continue to change over time. In North America, the incidences of metabolic dysfunction-associated steatohepatitis (MASH) and alcohol-associated liver disease (ALD) continue to increase as indications for LT [6]. In the United States, ALD is currently the most common indication for LT, followed by MASH, while in Canada, the most common indications for LT are HCC followed by ALD, representing a shift from hepatitis C virus (HCV) in both countries before the direct-acting antiviral (DAA) era [7,8,9]. Indications for LT in Europe are most frequently due to ALD, replacing HCV after the significant success of direct-acting antiviral therapies for HCV [2]. In Latin America, similarly, the most common indications for LT have become MASH and ALD, followed by HCV [10,11]. In Southeast Asia, Africa, and East Mediterranean regions, viral hepatitis due to hepatitis B virus (HBV) and HCV remain predominant, and related HCC is the most common indication for LT [2]. Across the world, the most common indication for pediatric LT is biliary atresia, which is responsible for approximately half of all pediatric LTs [12].

## 3. Geographic Dispersion of Living Donor Liver Transplant and Deceased Donor Liver Transplant

**Figure 1:**
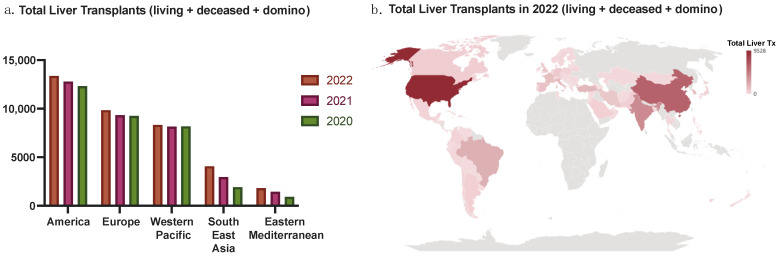
Total LTs in 2020, 2021, and 2022 based on geographical region (**a**), and total LTs in 2022 in the world (**b**). Data are based on the Global Observatory on Donation and Transplantation (GODT) data, produced by the WHO-ONT collaboration [4].

**Figure 2:**
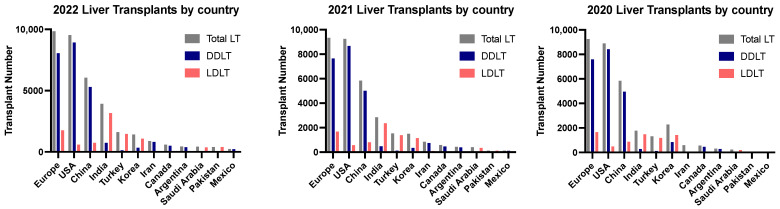
Geographical distribution of DDLT and LDLT activity, by various countries, in 2020, 2021, and 2022. Data are based on the Global Observatory on Donation and Transplantation (GODT) data, produced by the WHO-ONT collaboration [4].

There is a sizeable disproportionate allocation of LDLTs and DDLTs across the world. In countries such as India, Turkey, South Korea, Vietnam, Japan, and Kingdom of Saudi Arabia, LDLTs dominate most of the LT activity [13,14]. Pakistan, with around 500 LTs yearly and approximately 5000 patients needing LTs, solely depends on LDLT, as DDLT remains a challenge [15]. In North America, Latin America, Australia/New Zealand, and China, the focus and majority of activities are DDLT [13]. The disproportionate numbers between the Eastern Hemisphere and Western Hemisphere are vast. Approximately 90% of LT activity in the Eastern Hemisphere is LDLT, while nearly the opposite is true for the Western Hemisphere, for which 90% of LT activity is DDLT [13]. Despite this, LT rates in traditionally LDLT-dominant countries, specifically across Asia, are seeing a rise in DDLT, while countries with DDLT predominance continue to have relatively low rates of increases in LDLT [16,17]. Figure 1 shows the geographical distribution of LT activity by region, and Figure 2 shows the DDLT and LDLT activity by various countries in 2020, 2021, and 2022. In addition, we discuss some of the countries across the globe with high per million population LT rates below.

## 4. Living Donor Liver Transplant

LDLT was initially developed to increase organ availability due to scarce deceased organs; however, it later became the primary form of LT in many countries [18]. Though the difference in the Eastern and Western definitions grossly correlates with a difference in LDLT versus DDLT, LDLT is the predominant modality employed in many Asian countries, such as India, Pakistan, Japan, and South Korea, while China primarily utilizes DDLT [13,15]. Similarly, other countries, including Algeria, Tunisia, and Libya depend only on LDLTs due to sociocultural, economic, and religious reasons [19]. LDLT has been able to flourish through the development of different techniques, including right lobe grafting, dual grafting, acceptance of hepatitis B core antibody-positive patients, and tolerance of ABO incompatibility [20]. Those countries that employ LDLT also face numerous challenges in DDLT organ procurement, secondary to these same sociocultural reasons and various laws dictating the opportunity. Following the initiation of LDLT, these countries have continued to develop improved techniques, infrastructure, and policies to improve donor and recipient outcomes and provide safer LT [13]. Overall, in countries that predominantly perform LDLTs, strategies are employed to broadly maximize LDLT to counter the limited pool of deceased donor organs [18]. Ethically, concerns remain regarding the surgical procedure that a healthy donor must undergo, which, although rare, can lead to a significant number of complications, including liver failure and death, in addition to psychosocial complications [21,22].

### 4.1. India

LT in India is well-established but challenging to study due to the lack of an official centralized registry and database. India’s first successful LT was completed in 1998 in New Delhi, and currently over 1800 LTs are performed yearly, with 85% being LDLTs [23]. Most recent reports show MASH to be the main indication, followed by HCC and ALD, in India [24]. In 1994, India implemented the Human Transplant Act, legally accepting brain stem death and allowing for increased DDLTs. However, DDLT has not been widely utilized yet, and the most common form of LTs remain LDLTs due to multiple reasons, including the lack of an organized system for the identification and distribution of deceased organs, and sociocultural beliefs [24,25].

### 4.2. South Korea

The first LT in South Korea was performed in 1988 via DDLT, and the first LDLT was completed in 1994. Since then, South Korea established the Korean Network for Organ Sharing in 1999, which is responsible for managing the waitlist, organ allocation from deceased donors, and regulating and overseeing organ transplantation activities [26,27]. ALD prevalence is increasing in South Korea, and it was the most common etiology of cirrhosis in 2017, replacing HBV; and since 2013 rates of LT has been gradually increasing for ALD [28,29]. In 2020, there were 56 LT centers, with 41 centers performing both DDLTs and LDLTs, 5 centers strictly performing DDLTs, and 10 centers performing only LDLTs; additionally, the top 5 centers performed 974 cases, 60.3% of the national total LT activity [21]. The rate of DDLTs has shown an upward trend in South Korea over the past decade from 20% in 2010 to 34.5% in 2016, with a decrease in rates to 25% in 2017 [27]. There is a vastly low pool of brain-dead organ donors in South Korea, 8.7 per one million people in 2019, versus other countries that perform a significantly higher percentage of DDLTs, such as the United States, which had 36 per one million people in the same year [29]. The overall one-year survival of LTs performed in South Korea has been improving with a 3-year survival of 83.7% for LDLT and 80.9% for DDLT, and a 5-year survival of 72.3% for LLDT and 68.9% for DDLT [27].

### 4.3. Turkey

In Turkey, the first LT was performed in 1988, and the first successful partial LDLT was performed in 1990 [30,31]. In Turkey, there are more than 45 LT centers, and Turkey is one of the leading countries in LDLT in the world [30,32,33]. The most frequent indication for LT in adults is chronic viral hepatitis due to HBV and HCV, representing roughly one third of LTs, followed by ALD and MASH [34]. In line with trends observed in other countries, a shift in LT indications has been seen, with a transition from chronic viral hepatis to MASH and ALD in Turkey [34]. At Ege University, one of Turkey’s leading LT centers, from 1994–2017, 1001 LTs were performed (43% DDLTs, 57% LDLTs) [35]. This trend was noted by the Ministry of Health, outlining that from 2002–2013, 6091 LTs were completed, with 66% being LDLTs and 34% being DDLTs [30,31]. In 2019, a total of 1776 LTs (80.3% LDLTs) were performed in Turkey [32]. The Ministry of Health initially established the national organ-sharing program in 1989, and later the National Coordination Centers in 2001 for the allocation of deceased donors, which is now implemented in nine centers across Turkey in an attempt to grow the organ donation pool. In addition, surgical techniques to maximize donor organ and recipient matches have been performed in Turkey. In Malatya, the Inonu Liver Transplantation Institute has performed multiple complex living-donor–recipients paired exchanges, with the most complex being six simultaneously paired exchanges, and published reports on three and five LDLTs with successful outcomes [36].

### 4.4. Kingdom of Saudi Arabia

Within the Kingdom of Saudi Arabia (KSA), four centers offer LTs, and all four centers perform LDLTs, while only three perform DDLTs [37]. Each center that performs LTs has its own LT waiting list, with no national organ transplantation list [38]. The Saudi Center for Organ Transplantation oversees and manages the donation process while collaborating with individual centers [39]. Although HCV is the most common indication for LT, the proportion of patients with MASH is increasing [37].

The limited deceased donor organ pool has driven resources and the development of LDLT, which is offered to all patients requiring LT and has helped to mitigate the critical organ shortage [40]. KSA legally employs a brain death protocol for organ procurement, allowing for a staggering 90% of DDLTs in the Arab world performed in the KSA, with a total of 1251 deceased donor livers transplanted between 1990–2021 [41,42]. Uniquely, the government of the KSA financially compensates families who donate deceased donor livers as an expression of gratitude, which has raised ethical concerns [40].

### 4.5. Vietnam

In 2004, the first LT from a living donor was performed in Vietnam, and the first experimental LT from a brain-dead donor was performed in 2006 [43]. In 2010, the first successful LT from a brain-dead donor was conducted [43]. Since then, LT has gradually evolved in Vietnam, with advancements in surgical techniques, post-operative care, and organ procurement. The most common indications for LT in Vietnam are HBV and HCV infections, ALD, and HCC within Milan criteria or extended criteria [44]. Currently, several centers in Vietnam perform LTs, including major hospitals in Hanoi, Ho Chi Minh City (HCMC), and other regions. Some notable centers include 108 Military Central Hospital in Hanoi, Military Hospital 103 in Hanoi, Cho Ray Hospital in HCMC, and the University of Medicine and Pharmacy Medical Center in HCMC. These centers have established LT programs with experienced surgical teams, dedicated intensive care units, and comprehensive multidisciplinary support services. In Vietnam, LDLT is more common compared to DDLT, and this is mainly due to the shortage of deceased organ donors and the cultural acceptance of living organ donation among Vietnamese families [44]. Overall, LT in Vietnam has made significant progress over the years, providing life-saving treatment options for patients with end-stage liver disease. However, challenges such as organ shortage, access to healthcare services, and post-operative care standards continue to be areas of focus for further improvement.

### 4.6. Japan

As of 2017, a total of 9292 LTs at 67 transplant centers have been performed in Japan, including 447 DDLTs and 8795 LDLTs [45]. In Japan, the majority of LTs are LDLTs, and the most common indications for LT are cholestatic liver diseases followed by neoplastic diseases [45]. Similar to other countries, the rates of viral hepatitis is decreasing, while ALD is increasing. The revised Organ Transplant Law was made effective in 2010, allowing organ procurement from brain-dead donors with family consent, in an effort to increase the number of deceased organs [14]. However, the rates of DDLT have not significantly increased after this law, and a continuous focus for increasing the rates of DDLTs is ongoing [14].

## 5. Deceased Donor Liver Transplant

DDLT is the most common type of LT in many countries, such as the United States, Canada, the majority of European countries, China, Brazil, Australia, and New Zealand. Many Western countries utilize a large-scale organ donation infrastructure for the procurement of organs. This infrastructure typically includes rigorous evaluation, standardized resource allocation, and a reliable procurement process [46]. Counter to some LDLT-dominating countries, DDLT societies often employ laws that encourage deceased organ donation and honor the autonomy of individuals to make their own decisions regarding being organ donors, some even employing “opt-out” methods to set the default status for citizens to organ donors [47]. Several different bodies normalize and encourage organ donation through multiple media in these countries, working to raise awareness, knowledge, and contributions for organ donation [48]. The most significant benefit of DDLT is its extensive organ donor pool, providing further access to more people in situations where donor organs limit LDLT. Several developed techniques and protocols in Western countries have played a prominent role in the development and success of DDLT, similarly to how LDLT techniques have grown [49].

### 5.1. China

The first LT was completed in China in 1977, and in the 1990s the nation underwent a rapid growth and development for LTs; and as of 2021, 109 centers performed LTs in China [50]. The most frequent indication for LT in China is HBV, followed by HCC and ALD [50]. The China Liver Transplant Registry was established in 2005, recording and tracking almost all LT data in China; then, in 2013, the China Organ Transplant Response System was established as the sole organ allocation system, forbidding any LTs outside of the system [50]. Beginning in 2015, all LTs completed in China were mandated to be via DDLT or familial LDLT, and the country no longer permitted organ procurement from executed prisoners [50]. Standing apart from several of its geographical neighbors, China predominantly performs DDLT rather than LDLT despite significant advancements in LDLT [5,51].

### 5.2. Brazil

Brazil performs a large, growing number of LTs each year, predominantly DDLTs, and performs approximately 2/3 of all LTs in Latin America, with 78 active transplant centers [11]. In 2019, 2245 LTs were performed in Brazil, and of those, 2087 were via DDLTs and 158 were LDLTs, outlining the historical trend present each year [52]. Concerns over living donor morbidity and mortality initially decreased the LDLT rates following a surge in 2005 to 25% [53]. In 2015, there were 56 centers performing LTs, disproportionately centered in the Southeast and Southern regions in Brazil. The most common indication for being on the LT waitlist from 2010–2015 in a representative state of Brazil was viral hepatitis, which constituted 35% of the total LTs, followed by ALD at 11% [53]. Since 1997, the Brazilian Organ Transplantation Association has published annual reports regarding organ donation, providing a wealth of data regarding organ transplantation [54]. As of 2016, there were 67 organ procurement agencies in Brazil, covering all states [53]. The monetary support for LTs comes from the Brazilian Public Unified Health System, which funds 95% of all LTs and provides free and open LTs to Brazilian citizens.

### 5.3. Australia and New Zealand

Australia and New Zealand perform LTs through a platform of public funding via six LT centers and utilizes a shared database [55]. From 1985 until December 2014, 3700 adult LTs were performed in Australia and New Zealand, with the most common primary disease being HCV followed by ALD, HBV, and MASH [55,56]. By the end of 2014, only 88 LDLTs had been performed in four centers in Australia and one center in New Zealand, primarily in the pediatric population [55]. Demand is expected to continue to rise with the increased incidence of diabetes mellitus, obesity, and metabolic syndrome, and the projected increase in MASH [56,57].

### 5.4. United States

The United States performs most of the world’s LTs, with 9234 LTs completed in 2021, 93.8% via DDLTs and 6.2% via LDLTs [8]. There is a rigorous government agency in place for organ transplantation in the United States, established under the National Organ Transplant Act in 1984, which is the Health Resource and Service Administration; and it provides oversight and recovery through the Organ Procurement and Transplant Network (OPTN), which is operated by a private organization, The United Network for Organ Sharing (UNOS). In the United States, ALD remains the primary indication for nearly 40% of all LTs, more than double the amount compared to 10 years prior [7]. The second most common indication is MASH, followed by cryptogenic cirrhosis and HCC [8]. Some published reports have been advocating for the increased utilization of LDLTs within the United States, reporting some superior outcomes and lower resource utilization, which has yielded an expansion on a national level [58]. In 2010, 28 LT centers were performing LDLTs, which increased to 43 in 2019; this corresponded to a near doubling of the LDLT frequency, with a total of 2566 LDLTs during the same period [59]. For DCD LT, a substantial variability in terms of the practice and utilization of DCD donors exists. The majority of DCD LTs is utilized in few centers with high LT volumes, and the graft survival has improved in more recent years compared to early years in the United States [60,61,62]. Machine perfusion, however, has been adapted at a slower rate in the United States compared to Europe, as the first Food and Drug Administration (FDA) approval for an ex situ perfusion device was established on September 2021 [63]. As the FDA approval is relatively recent, no large clinical trials or cohort studies for graft and patient survival are present for the application of normothermic machine perfusion in the United States.

### 5.5. Europe

The first LT attempt was made in 1964 in Europe, and after this LT has been widely accepted as a treatment modality [32]. The main indications for LT in many European countries are HCV, ALD, and HCC. Overall, the rates of HCV are decreasing the success and availability of DAAs [2]. LT is organized and executed by national transplant organizations in many European countries. In addition, transnational organizations, such as Eurotransplant, Scandiatransplant, and South Alliance for Transplantation oversee organ allocation among their country members. Eurotransplant was founded in 1967 and incudes Austria, Belgium, Crotia, Germany, Hungary, Luxembourg, the Netherlands, and Slovenia. In Eurotransplant, all members list their patients into a central international waitlist, and individual countries follow their own policies. If no match is available on the national waiting list, the donor is registered at Eurotransplant. Scandiatransplant is another transnational organization that was founded in 1969, and it includes Denmark, Finland, Iceland, Norway, Sweden, and Estonia. It uses a centralized organ allocation policy based on waiting time, and, similar to Eurotransplant, all centers list their patients in a central waitlist. South Alliance for Transplantation was founded in 2012 and includes Czechia, France, Italy, Portugal, Spain, and Switzerland, and its member countries follow their own national transplantation policies. In 2018, a total of 9858 LTs were performed in Europe [32]. Countries including Croatia, Belgium, and Spain have high per million population LT rates compared to lower rates in Romania, Bulgaria, and Greece [32]. The majority of LT activity is DDLT and, with the exception of Turkey, LDLT contributes to <5% of all LTs in Europe [64,65]. In addition, DCD contributes to as much as 20% of all donor pools in certain European countries; however, significant heterogeneity exists for the utilization, practice, and application of modern perfusion technologies for DCD LT [66]. DCD LT rates are particularly high in Belgium, France, Italy, Spain, and Switzerland in Europe compared to other countries [67]. Machine perfusion is used more commonly in Europe compared to the United States [63].

## 6. Outcomes after Liver Transplantation

Transplant outcomes, either for DDLTs or LDLTs, are challenging to compare across nations as there is no international database to track patient outcomes. In some studies, LDLT has been reported to have more favorable outcomes immediately following LT, possibly decreasing the length of hospital admissions, blood transfusions, and renal replacement therapy [58]. Another study on a matched cohort comparison reported that LDLT had a higher overall rate of perioperative complications, mainly due to biliary complications, compared to DDLT, but 3- and 5-year graft and patient survivals were similar [68]. Multiple other studies also reported no significant differences in graft loss and patient survival in LDLT compared to DDLT [69,70]. However, another meta-analysis conducted in the United States reported a lower mortality at 1-, 3-, 5-years post-LT, a lower risk of rejection, but similar graft survival [71]. Despite this, through the advancement of surgical techniques and improvements in medical management, the risk of donor morbidity and mortality has decreased [72].

## 7. Conclusions

As the demand for liver increases, healthcare providers have found ways to improve the practice of LT through advances in procurement methods, surgical techniques, and medical management. The higher rates of LDLT in Asian countries are multifaceted and stem from numerous influences, including infrastructure, surgical techniques, laws, donor rights advocates, and belief systems. In many Western countries, these same entities drive DDLT, with infrastructure focusing on organ procurement and laws and belief systems focusing on autonomy organ donation. Moving forward, complementing DDLT with LDLT to achieve the most favorable outcomes remains important.

## Data Availability

Data sharing is not applicable. No new data were created or analyzed in this study. Data sharing does not apply to this article.

## Abbreviations

ALD	alcohol-associated liver disease
DDLT	deceased donor liver transplant
GODT	Global Observatory on Donation and Transplantation
HBV	hepatitis B virus
HCC	hepatocellular carcinoma
HCV	hepatitis C virus
LT	liver transplantation
LDLT	living donor liver transplant
MASLD	metabolic dysfunction-associated steatotic liver disease
FDA	Food and Drug Administration
DCD	Donation after circulatory death
MASH	metabolic dysfunction-associated steatohepatitis
